# Knowledge-Driven Approaches to Create the MTox700+ Metabolite Panel for Predicting Toxicity

**DOI:** 10.1093/toxsci/kfac007

**Published:** 2022-01-30

**Authors:** Elena Sostare, Thomas N Lawson, Lucy R Saunders, John K Colbourne, Ralf J M Weber, Tomasz Sobanski, Mark R Viant

**Affiliations:** Michabo Health Science, University of Birmingham Enterprise, Birmingham Research Park, Birmingham B15 2SQ, UK; Michabo Health Science, University of Birmingham Enterprise, Birmingham Research Park, Birmingham B15 2SQ, UK; School of Biosciences, University of Birmingham, Birmingham B15 2TT, UK; Michabo Health Science, University of Birmingham Enterprise, Birmingham Research Park, Birmingham B15 2SQ, UK; School of Biosciences, University of Birmingham, Birmingham B15 2TT, UK; School of Biosciences, University of Birmingham, Birmingham B15 2TT, UK; European Chemicals Agency (ECHA), 00150 Helsinki, Finland; Michabo Health Science, University of Birmingham Enterprise, Birmingham Research Park, Birmingham B15 2SQ, UK; School of Biosciences, University of Birmingham, Birmingham B15 2TT, UK

**Keywords:** systematic review, metabolite, metabolomics, biomarker, adverse outcome, molecular pathway

## Abstract

Endogenous metabolite levels describe the molecular phenotype that is most downstream from chemical exposure. Consequently, quantitative changes in metabolite levels have the potential to predict mode-of-action and adversity, with regulatory toxicology predicated on the latter. However, toxicity-related metabolic biomarker resources remain highly fragmented and incomplete. Although development of the S1500+ gene biomarker panel has accelerated the application of transcriptomics to toxicology, a similar initiative for metabolic biomarkers is lacking. Our aim was to define a publicly available metabolic biomarker panel, equivalent to S1500+, capable of predicting pathway perturbations and/or adverse outcomes. We conducted a systematic review of multiple toxicological resources, yielding 189 proposed metabolic biomarkers from existing assays (BASF, Bowes-44, and Tox21), 342 biomarkers from databases (Adverse Outcome Pathway Wiki, Comparative Toxicogenomics Database, QIAGEN Ingenuity Pathway Analysis, and Toxin and Toxin-Target Database), and 435 biomarkers from the literature. Evidence mapping across all 8 resources generated a panel of 722 metabolic biomarkers for toxicology (MTox700+), of which 462 (64%) are associated with molecular pathways and 575 (80%) with adverse outcomes. Comparing MTox700+ and S1500+ revealed that 418 (58%) metabolic biomarkers associate with pathways shared across both panels, with further metabolites mapping to unique pathways. Metabolite reference standards are commercially available for 646 (90%) of the panel metabolites, and assays exist for 578 (80%) of these biomarkers. This study has generated a publicly available metabolic biomarker panel for toxicology, which through its future laboratory deployment, is intended to help build foundational knowledge to support the generation of molecular mechanistic data for chemical hazard assessment.

The measurement of endogenous metabolite levels to predict toxicity, whether by untargeted metabolomics or targeted metabolite assays, has received increasing attention over the last decade (Johnson *et al.*, 2016; Kamp *et al.*, 2012; Ramirez *et al.*, 2013; Sperber *et al.*, 2019; Viant *et al.*, 2019). Metabolic measurements describe the most downstream molecular phenotype, providing insights into a substance’s mode-of-action (MoA) and, critically, biomarker profiles that are strongly associated with adverse (or apical) endpoints upon which regulatory toxicology is predicated (Hines *et al.*, 2010; Taylor *et al.*, 2018). Some individual metabolic biomarkers are already measured as part of international regulatory test guidelines, such as triiodothyronine and thyroxine hormones, to predict thyroid toxicity in rodent repeated-dose 90-day studies (OECD 2018). Other metabolic biomarkers, discovered via untargeted metabolomics, have been deployed in targeted screening assays, including ornithine and cystine for predicting developmental toxicity (Zurlinden *et al.*, 2020). The regulatory application of a broader panel of more than 200 metabolic biomarkers, predictive of multiple MoAs, has been extensively demonstrated by BASF, in particular for category formation to support read-across (Kamp *et al.*, 2012; Mattes *et al.*, 2014; Sperber *et al.*, 2019; Van Ravenzwaay *et al.*, 2015). Although these examples collectively demonstrate the value of metabolic biomarkers in regulatory toxicology, their implementation remains limited. This is in part because toxicity-related metabolic biomarker resources for human health remain highly fragmented and incomplete.

In 2013, the U.S. National Toxicology Program (NTP) launched an initiative that utilized data- and knowledge-driven approaches to create a human transcriptomics biomarker panel, the S1500+ targeted gene set, to enable cost-effective, high-throughput measurements that are predictive of pathway perturbations (Mav *et al.*, 2018). By establishing this gene biomarker panel on the TempO-Seq gene expression platform (Yeakley *et al.*, 2017), the application of targeted transcriptome profiling in toxicology has increased substantially and rapidly (Bushel *et al.*, 2018). In contrast, no equivalent study has been reported that interrogates information from multiple resources to derive a panel of metabolic biomarkers that have the potential to predict pathway perturbations and/or adversity. Yet, there are several justifications to creating such a panel, not least as it could circumvent the challenge of metabolite identification that plagues untargeted metabolomics studies in toxicology. In addition, it could improve harmonization of the analytical approaches used and accelerate the generation of informatics resources that describe levels of identified metabolites in the context of predicting toxicity endpoints. Such resources are sorely lacking in metabolomics compared with transcriptomics (Igarashi *et al.*, 2015; Lamb *et al.*, 2006; Richard *et al.*, 2016), arguably another factor underlying the limited implementation of metabolomics into regulatory toxicology. Defining robust mechanistic associations between metabolic biomarkers and adverse outcomes (AOs) would increase the acceptance of this New Approach Methodology into hazard assessment frameworks.

The overall aim of this work was to define for the first time a metabolic biomarker panel for toxicology, similar to the definition of the S1500+ gene biomarker panel. This was achieved by mining multiple toxicological resources—including existing multiplexed molecular assays, databases, and the literature—to identify a panel of human health-relevant metabolites associated with disease, toxicity, and other AOs in humans. The first objective was to create a universal list of detectable human metabolites (“metabolite master list [MML]”), derived from the Human Metabolome Database (HMDB), for facile and rigorous filtering of metabolites through all subsequent phases in the project. Next, multiple toxicological resources containing information on metabolic biomarkers were identified, and data were extracted from each of these, including 3 multiplexed assays, 4 databases, and the published literature. To maximize confidence in the predictivity of these biomarkers and hence their utility in regulatory decision making, further information was gathered to assign one or more disease and/or AOs to each metabolite. To help provide guidance on the context of use of the metabolic biomarkers, the type(s) of samples in which the biomarkers have previously been measured was collected. Furthermore, pathway sources were interrogated to link the biomarkers to molecular mechanisms, allowing pathway complementarity to the S1500+ gene biomarker panel to be assessed. The availability of analytical assays and reference standards for each metabolic biomarker was investigated to assess the community’s capability to measure the biomarkers routinely. This first version of the proposed metabolic biomarker panel for predictive toxicology is termed MTox700+, which is made available at https://michabo.co.uk/resources/mtox (version 1, updated on 12/01/2022).

## MATERIALS AND METHODS

###  

#### Creation of MML From HMDB and MetaboLights

**Figure 1.**
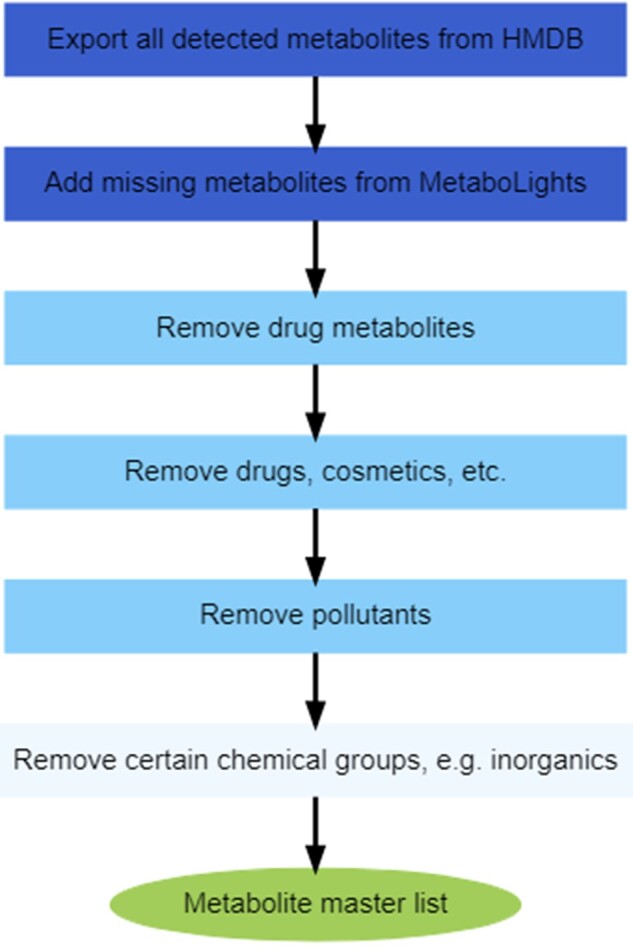
Workflow to create the metabolite master list (MML). The first 2 boxes describe the importing of metabolites from the HMDB (Human Metabolome Database; Wishart *et al.*, 2007) and MetaboLights data repository (Haug *et al.*, 2013). The third to fifth boxes represent the filters that were used to refine the metabolite list based on HMDB ontology, and in the sixth box further filters based on chemical ontology were applied. This process yielded the MML of detectable human-relevant metabolites.

An “MML” of detectable human-relevant metabolites was created based on the HMDB (Wishart *et al.*, 2007; version 4.0 [Wishart *et al.*, 2018], release date—July 9, 2018) and MetaboLights data repository (Haug *et al.*, 2013; downloaded on February 29, 2020; identifiers were converted from ChEBI to HMDB using The Chemical Translation Service—CTS [Wohlgemuth *et al.*, 2010]). The MML was created for multiple reasons: to ensure consistency in naming metabolites throughout the study; to ensure consistency in filtering across each of the individual metabolite resource lists; to facilitate removal of all drugs, solvents, and other exposure-related chemicals from these lists; and to assess whether metabolites are analytically detectable according to HMDB (version 4.0)/MetaboLights, as only detectable metabolites were listed in the MML. To remove several unwanted groups of chemicals from the MML, various types of ontology were used to create ontology filters, which were applied to the combined HMDB/MetaboLights MML as described in Figure 1. These ontology filters used both the HMDB 4.0 hierarchical ontology and the chemical ontology from ClassyFire (Djoumbou Feunang *et al.*, 2016). First, drug metabolites were removed using the “biological role” HMDB ontology filter. Then drugs, personal care products, cosmetics, and laboratory chemicals were removed using the “industrial application” HMDB ontology filter. Environmental pollutants/contaminants were removed using the “environmental role” HMDB ontology filter. In addition, chemical ontology filters were applied to remove any remaining drug and food exposure-related chemical groups, which comprised the removal of inorganics, organometallic compounds, alkaloids and derivatives, hydrocarbons, organic 1,3-dipolar compounds, organic polymers, organohalogen compounds, biphenyls, naphthalenes, fluorenes, phenanthrenes and derivatives, tetralins, organic dithiophosphoric and thiophosphoric acids, oxepanes, isothiocyanates, sulfoxides, flavonoids, isoflavonoids, phenylpropanoic acids, and diarylheptanoids.

#### Extraction, Filtering, and Merging of Metabolite Resource Lists to Create Proposed Metabolic Biomarker Panel—MTox700+

**Figure 2.**
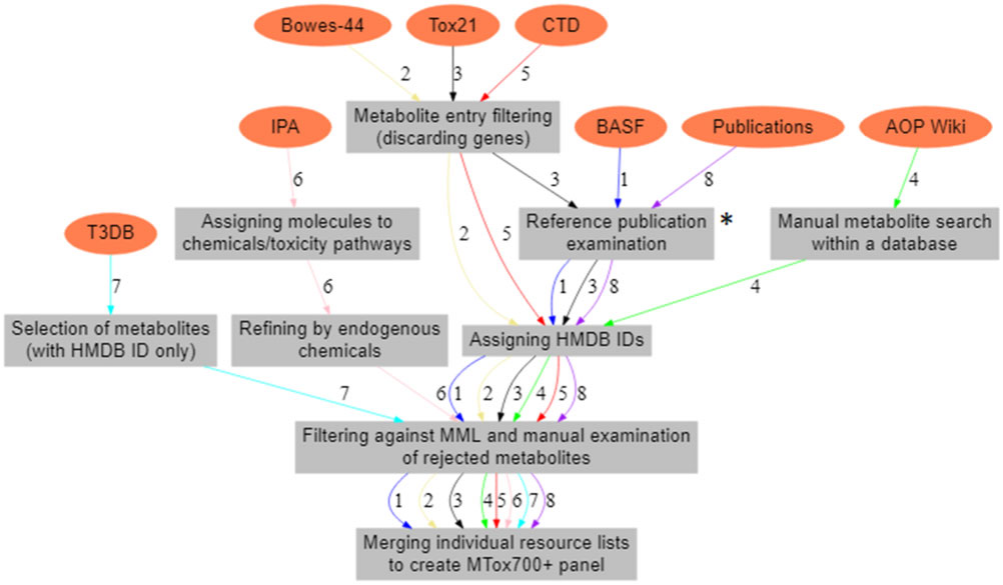
Workflow to create 8 individual metabolite resource lists. These include 3 multiplexed assay lists—BASF metabolic biomarker panel (workflow 1, labeled on the arrows), Bowes-44 pharmacological biomarker panel (workflow 2), and Tox21 assays (Toxicology in the 21st Century, workflow 3); 4 databases—AOP (Adverse Outcome Pathway Wiki, workflow 4), CTD (Comparative Toxicogenomics Database, workflow 5), IPA (Ingenuity Pathway Analysis, workflow 6), and T3DB (Toxin and Toxin-Target Database, workflow 7); and the list of metabolites from the published literature (workflow 8). *Reference publications were examined in the resource or the Abstract Sifter.

Eight existing toxicological resources were selected for interrogation, including 3 multiplexed assays, 4 databases, and the published literature. A short description of each resource, together with a justification for its inclusion, is presented below. For each resource, a list of metabolites was either directly extracted or the resource was searched manually, filtering was then applied to ensure only high-data quality was retained, and a metabolite resource list was produced; see Figure 2 for an overview of the steps.

##### Multiplexed assays

“BASF” developed a metabolic biomarker panel for toxicology studies of rat, which has been used to create the MetaMap Tox database containing the responses of the plasma metabolome to more than 1000 chemicals (Sperber *et al.*, 2019). The database associates changes in metabolite levels with rodent toxicity outcomes and hence the metabolic biomarker panel has high relevance to the current study. The BASF assay comprises 202 metabolic biomarkers (most are identified, some remain unknown) and was imported from Sperber *et al.* (2019). Where possible, HMDB IDs were assigned to metabolites using CTS.

“Bowes-44 (or SafetyScreen-44)” is a pharmacological biomarker panel originally developed by a consortium of pharmaceutical companies (Bowes *et al.*, 2012) and now commercially available via Eurofins. The 44 targets represent a minimal panel that provides a broad early assessment of potential human health hazards in drug development. It was selected for this study due to its high toxicological relevance and the availability of the assay. The panel currently comprises 47 measured endpoints. All gene biomarkers were removed and HMDB IDs were assigned to the metabolite entries using CTS.

“Toxicology in the 21st Century (Tox21)” program is a joint initiative between 3 U.S. agencies, the National Center for Advancing Translational Sciences, NTP, and the Food and Drug Administration (Thomas *et al.*, 2018). Tox21 aims to improve toxicity assessment methods and provide a rapid and robust assessment of a chemical and its potential human health effects. Due to the high human toxicological relevance of the biomarkers measured in the Tox21 assays, they were considered for inclusion in this study. The Tox21 assay list was examined for metabolites, all gene biomarkers were removed, and the remaining entries containing reference publications were curated. Metabolites were then assigned HMDB IDs using CTS.

##### Databases

“Adverse Outcome Pathway (AOP) Wiki” is an open-source toxicological database, managed by the OECD, that structures data around a framework connecting a molecular initiating event to an AO (Ankley *et al.*, 2010). The AOP Wiki organizes the knowledge of toxicological perturbations into base units—key events (KEs) and KE relationships—extensively describing the underlying biological and experimental information. Currently, there are more than 260 AOPs in this knowledge base. Data within the AOP Wiki were selected for this study due to its focus on toxicological perturbations and the linkage of molecular KEs to AOs. A manual search for metabolites within all of the AOP KEs was performed. HMDB IDs were assigned to metabolites using CTS.

“Comparative Toxicogenomics Database (CTD)” is an open-source database, funded by the U.S. National Institute of Environmental Health Sciences, that focuses on the environmental exposures affecting human health (Davis *et al.*, 2019; Mattingly *et al.*, 2003). Although CTD primarily emphasizes the associations of gene biomarkers with chemicals and disease, it also contains some metabolic biomarker-chemical connections; hence, it is a further valuable resource for the current study. A matrix defining the toxicity-associated biomarkers, including genes, proteins, drugs, and metabolites, was exported from the CTD database. All gene and protein biomarkers were removed, as were all entries for which the detected “stressor” (drug/toxin) is the “marker.” HMDB IDs were assigned to the remaining metabolites using CTS.

“QIAGEN Ingenuity Pathway Analysis (IPA, QIAGEN Inc., https://digitalinsights.qiagen.com/qiagen-ipa, last accessed February 2, 2022)” is a commercial knowledgebase that was created by compiling a vast amount of information on molecular mechanisms associated with disease (primarily) and toxicology, in human, mouse, and rat (Krämer *et al.*, 2014). It encompasses over 7.8 million total findings and approximately 90 000 curated publicly available datasets, which can be used to predict potential therapeutic or toxicity targets, and drugs acting on those targets. With over 16 850 metabolites (including endogenous metabolites and xenobiotics), and containing a variety of metabolite associations with diseases, biological functions, and/or pathways, IPA is particularly effective at deriving toxicity pathway-associated metabolites and therefore of considerable value to this study. First, IPA was interrogated for any human-relevant metabolites that were associated with exposure to “Substances of Very High Concern” (SVHC; list obtained from European Chemical Agency’s website on August 8, 2019). An SVHC is a substance of particular concern for human health, including carcinogens, mutagens, reproductive toxicants, and chemicals that are persistent, bioaccumulative, and toxic. This involved searching for the SVHCs and submitting the findings to the “Pathway builder” module to assign any molecular associations. Second, a search to find relevant metabolites associated with IPA’s toxicity pathways was conducted. The metabolite lists from both strategies were then refined by keeping only “endogenous chemicals.”

“Toxin and Toxin-Target Database (T3DB, or Toxic Exposome Database)” is an open resource developed by The Metabolomics Innovation Center, Canada, that focuses on providing mechanisms of toxicity and target molecules for each toxin (Lim *et al.*, 2010). It is closely linked to the HMDB, Small Molecule Pathway Database (SMPDB), and PathBank DB. The T3DB contains 42 374 toxin-toxin target associations (accessed on January 6, 2020). Due to its large and broad information content (chemical information, ontologies, origin, pathways, exposure, health effects, etc.), and despite regarding metabolites as “toxins,” T3DB contains considerable relevant data on metabolites and therefore was selected for inclusion in this study. The initial matrix with abundant toxicological information was sourced from T3DB “toxin” matrix file. Metabolites with HMDB IDs (which were already present in this matrix) were selected for further curation.

##### Published literature

To provide a broader strategy to identify metabolic biomarkers of toxicity beyond those derived from the 3 multiplexed assays and 4 database resources, an extensive search of the published literature was undertaken. This strategy also helped to ensure the latest scientific discoveries were included in the proposed metabolic biomarker panel. With our focus on biomarkers for human health, publication abstracts in the PubMed repository (Sayers *et al*. 2022) were curated using Abstract Sifter (Baker *et al.*, 2017). Abstract Sifter is a Microsoft Excel-based application that was developed by the U.S. Environmental Protection Agency to enhance existing search capabilities of PubMed. It allows keyword searching, effective organization and visualization of publication lists, and ranking of the processed references. Our structured search was performed using the query “metabolite and toxicity and biomarker.” All publications meeting these search criteria, but which contained only gene or protein biomarkers, provided no evidence of substance toxicity, or were nonmammalian, were considered as false positives and therefore rejected. Publications that contained metabolic biomarkers of exposure (such as drugs and drug metabolites) were also rejected. If the abstract referred to nonspecific or partial metabolite names, the full text of the publication was then examined. The metabolite resource list from the published literature was manually prepared using the results from the Abstract Sifter. Finally, HMDB IDs were assigned to the metabolites using CTS.

Following the export of 8 individual resource lists, above, each list was filtered against the MML to ensure it contained only biological, human-relevant metabolites with unique HMDB IDs assigned to each metabolite (Figure 2). Any metabolites that were found to occur in a resource list, but not in the MML, were manually re-inspected before deciding whether to accept or remove them from the resource list. This was necessary because some biologically relevant metabolites were listed as “undetected” in HMDB, but are “analytically detectable” according to the other toxicological resources that were examined (assays, databases, or publications), and these metabolites were therefore retained in this study. Furthermore, all metabolite entries were manually reviewed to identify any remaining errors, ie, to ensure that all metabolites were of biological origin by ensuring all drugs, drug metabolites, environmental pollutants/contaminants, and laboratory chemicals were removed. In addition, due to varying levels of confidence in metabolite identification, we derived metabolite identification levels (where possible) from each of the resources; ie, as based upon the Metabolomics Standards Initiative (MSI) guidelines (Sumner *et al.*, 2007)—MSI level (1) Identified compounds, MSI level, (2) Putatively annotated compounds, MSI level, (3) Putatively characterized compound classes, and MSI level (4) Unknown compounds. The type of sample (eg, type of tissue, biofluid, and/or cells) in which each of the metabolites was measured (where the information was available) was also extracted.

The next step was to merge all 8 resource lists to produce the proposed metabolic biomarker panel for toxicology—MTox700+. This was achieved through the use of HMDB IDs that were assigned to all metabolites in all of the resource lists.

#### Associating Disease and/or AOs With Each Metabolite in the Proposed MTox700+ Panel

Associations between each metabolite in the proposed MTox700+ panel with disease and/or AOs were derived using further data extracted from 4 AO sources—HMDB, IPA, AOP Wiki, and publications. Specifically, HMDB (version 4.0) contained metabolite-disease associations that were derived from the Online Mendelian Inheritance in Man (OMIM) database (Hamosh *et al.*, 2000). Using IPA, a “metabolomics core (enrichment) analysis” was conducted employing all of the metabolites in the MTox700+ panel as input, in order to extract “disease and biological function” associations for each metabolite. The AOP Wiki was manually searched to extract any relevant associations between metabolic KEs and AOs. Publications were examined in Abstract Sifter and AOs associated with the publications containing metabolites of interest were extracted manually.

#### Associating Molecular Pathways With Each Metabolite in the Proposed MTox700+ Panel

**Figure 3.**
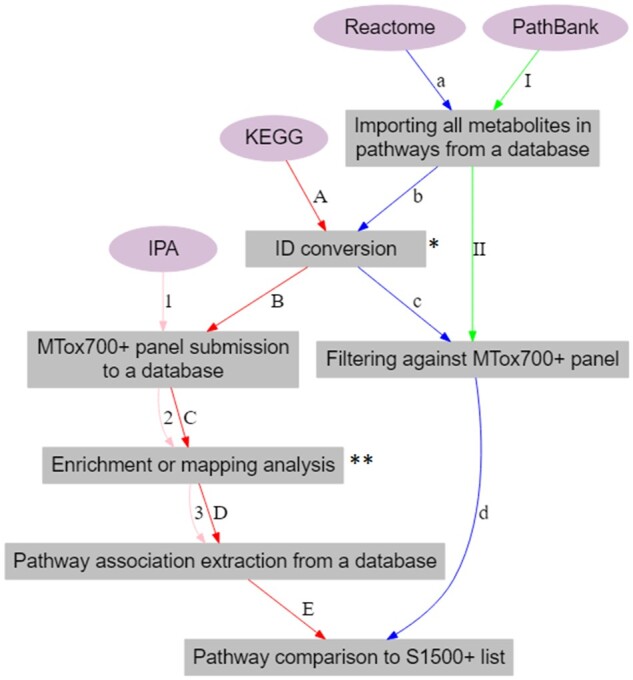
Workflow for connecting metabolites from the MTox700+ panel to molecular pathways. The pathway sources used include IPA (Ingenuity Pathway Analysis, 1–3), KEGG (Kyoto Encyclopedia of Genes and Genomes, A–E), Reactome (a–c), and PathBank (I–II). *Human Metabolome Database (HMDB) IDs were converted to KEGG IDs in KEGG workflow and ChEBI IDs were converted to HMDB IDs in Reactome workflow. **Enrichment analysis was performed in IPA and metabolite mapping was performed in KEGG.

Molecular pathway associations with the metabolites in the proposed MTox700+ panel were derived from 4 pathway sources (Figure 3)—IPA (Krämer *et al.*, 2014), Kyoto Encyclopedia of Genes and Genomes (KEGG; Kanehisa and Goto, 2000), Reactome (Joshi-Tope *et al.*, 2005), and PathBank (Wishart *et al.*, 2020).

In “IPA,” similar to the disease workflow, all of the MTox700+ panel metabolites were used as input and “metabolomics core (enrichment) analysis” was conducted to extract pathway associations for each metabolite.

KEGG and Reactome were selected due to their rich pathway content and S1500+ panel compatibility. “KEGG” (by Kanehisa Laboratories, Japan) is an open-source pathway database for understanding high-level functions and utilities of biological systems (cells, organisms, and ecosystems) from molecular-level information. To extract pathway associations from KEGG, identifiers from the MTox700+ panel were first converted from HMDB to KEGG using CTS (Wohlgemuth *et al.*, 2010). The metabolites were then submitted into KEGG Mapper and pathway-metabolite associations were derived from the database.

“Reactome” (developed by Ontario Institute for Cancer Research, New York University School of Medicine, European Molecular Biology Laboratory’s European Bioinformatics Institute, and Oregon Health & Science University) is an open source, manually curated, and peer-reviewed pathway database. It provides bioinformatics tools for the visualization, interpretation, and analysis of pathway knowledge to support basic and clinical research using omics data. Using Reactome, all of the pathway-metabolite associations were extracted, and identifiers were converted from ChEBI to HMDB using CTS. The metabolites with associations were then filtered against the MTox700+ panel metabolites yielding panel-specific pathway-metabolite associations.

“PathBank” (closely connected to SMPDB and HMDB, by The Metabolomics Innovation Centre, Canada) is an open-source interactive pathway database with more than 100 000 pathways. Its primary focus is metabolomics, and it therefore contains a set of unique pathways not found in other databases. Using PathBank, all the pathway-metabolite associations were imported, and the metabolites with associations were filtered against the MTox700+ panel yielding panel-specific pathway-metabolite associations.

For reliability of the interrogated pathways and consistency with the Tox21 S1500+ methodologies, after obtaining pathway association data from all 4 pathway sources, each pathway list was reviewed and only pathways with 3 or more participating metabolites (termed “reliable” molecular pathways) were retained. The list of metabolites associated with these reliable pathways was collated and described in the results.

Furthermore, the reliable molecular pathways (associated with metabolic biomarkers from the KEGG and Reactome databases) were compared with the pathways associated with the S1500+ gene biomarker panel (Mav *et al.*, 2018) to determine the overlap of pathways between the MTox700+ and S1500+ biomarker panels.

#### Associating Each Metabolite in the Proposed MTox700+ Panel With Analytical Assays and Reference Standards

The MTox700+ panel metabolites were assigned an analytical assay type based upon information from several resources: all 3 multiplexed assay resources (BASF, Bowes-44, and Tox21), 1 database resource (CTD), and publications. Specifically, analytical assay types for measuring metabolites of interest (such as Liquid Chromatography–Mass Spectrometry (LC-MS), Gas Chromatography–Mass Spectrometry (GC-MS), Nuclear Magnetic Resonance (NMR), etc.) were obtained from Sperber *et al.* (2019) for the BASF panel, from Bowes *et al.* (2012) for the Bowes-44 panel, and from Kleinstreuer *et al.*, (2011) for the Tox21 panel. Of the multiple databases interrogated in this project, only the CTD resource provided assay information, which was extracted from the “exposure events” file, and then nonanalytical methods such as questionnaires, predictions, and computational analyses were removed from the final assay list. Analytical assay types were also extracted from publications, which were manually curated in Abstract Sifter (Baker *et al.*, 2017); the full text was reviewed if the assay was not provided in the abstract. All assays were then merged into a single list, providing an overview of how each panel metabolite can be measured.

The availability of reference standards was first assessed by checking against 2 large metabolomics libraries—IROA Technologies Mass Spectrometry Metabolite Library of Standards and MetaSci COMPLETE Metabolite Library, followed by a manual search for the remaining metabolites from other vendors including ABI Chem, Avanti, CaymanChem, Enzo Life Sciences, MedChemExpress, MolPort, Sigma Aldrich (Merck), TargetMol, and Thermo Fisher.

#### Ranking the Importance of Metabolites in the Proposed MTox700+ Panel

To aid the ranking of metabolites in the MTox700+ panel based on their perceived importance in toxicological responses, several criteria were developed. Three separate scores were assigned to each metabolite in the panel based upon each of 3 properties: (1) total coverage in toxicological resources, AO sources, and pathway sources, (2) pathway consistency with the S1500+ gene panel, and (3) measurement feasibility.

The first ranking score for each metabolite was based on its “total coverage in toxicological resources, AO sources, and pathway sources”, considering whether the metabolite featured in the original 8 resources (described in Extraction, Filtering, and Merging of Metabolite Resource Lists to Create Proposed Metabolic Biomarker Panel—MTox700+ section; score of 1–8), whether the metabolite was present in the sources of AO information (described in Associating Disease and/or AOs With Each Metabolite in the Proposed MTox700+ Panel section; score of 0–4), and whether the metabolite was present in the pathway sources (described in Associating Molecular Pathways With Each Metabolite in the Proposed MTox700+ Panel section; score of 0 to 4). The total coverage score was calculated as: Score_(total coverage %)_ = [(toxicological resource count/8*100 + AO source count/4*100 + pathway source count/4*100)]/3. Finally, the total coverage score was categorized into 3 levels—low (score below 33%), medium (score 33–66%), and high (score above 66%).

The second ranking score for each metabolite described the “pathway consistency with the S1500+ gene panel,” either as consistent (score of 1) or not (0), with consistency defined as the metabolite being present in a reliable molecular pathway (see definition in Associating Molecular Pathways With Each Metabolite in the Proposed MTox700+ Panel section), where that pathway is also measured by genes in the S1500+ panel.

Finally, the third ranking score was based on “measurement feasibility,” based on the availability of an analytical assay (score of 1, if available) and a reference standard (score of 1, if available); total score of 0–2.

## RESULTS

###  

#### Creation of MML From HMDB and MetaboLights

Multiple international data resources utilize their own identifiers for individual metabolites. Since the principal objective of this work was to integrate data from multiple toxicological resources, we first established a core list of consistently named metabolites against which we could map each of the metabolite resource lists of proposed biomarkers. The HMDB was selected as the primary source of metabolites and their identifiers for the master list as it is the most extensive human-relevant metabolite resource internationally. This was complemented by metabolites reported in the European Bioinformatics Institute’s MetaboLights database, the most extensive repository of experimental metabolomics data in Europe.

The HMDB unfiltered metabolite list contained 9052 detectable (quantified or nonquantified) metabolites. In addition, the MetaboLights database was imported to ensure that any (newly) detected metabolites missing from the HMDB were included, yielding 822 metabolites that could be assigned HMDB IDs (converted from ChEBI IDs using CTS). Of these, 121 were not present in the 9052 HMDB metabolite list, hence were added to form a master list with 9173 metabolites. After ontology filtering (Figure 1, boxes 3–6), 8658 metabolites remained (42 of those lacked any ontology terms, but were retained), forming the final MML, all with HMDB IDs (see Supplementary Material 1 MML and resource lists, tab “MML”).

#### Creation of Metabolite Resource Lists and Proposed Metabolic Biomarker Panel—MTox700+

To create the metabolite resource lists of proposed biomarkers, multiple existing toxicological resources were interrogated including 3 multiplexed assays—BASF, Bowes-44, and Tox21, 4 databases—AOP Wiki, CTD, IPA, and T3DB, and the published literature, as introduced in the Materials and Methods section.

**Figure 4.**
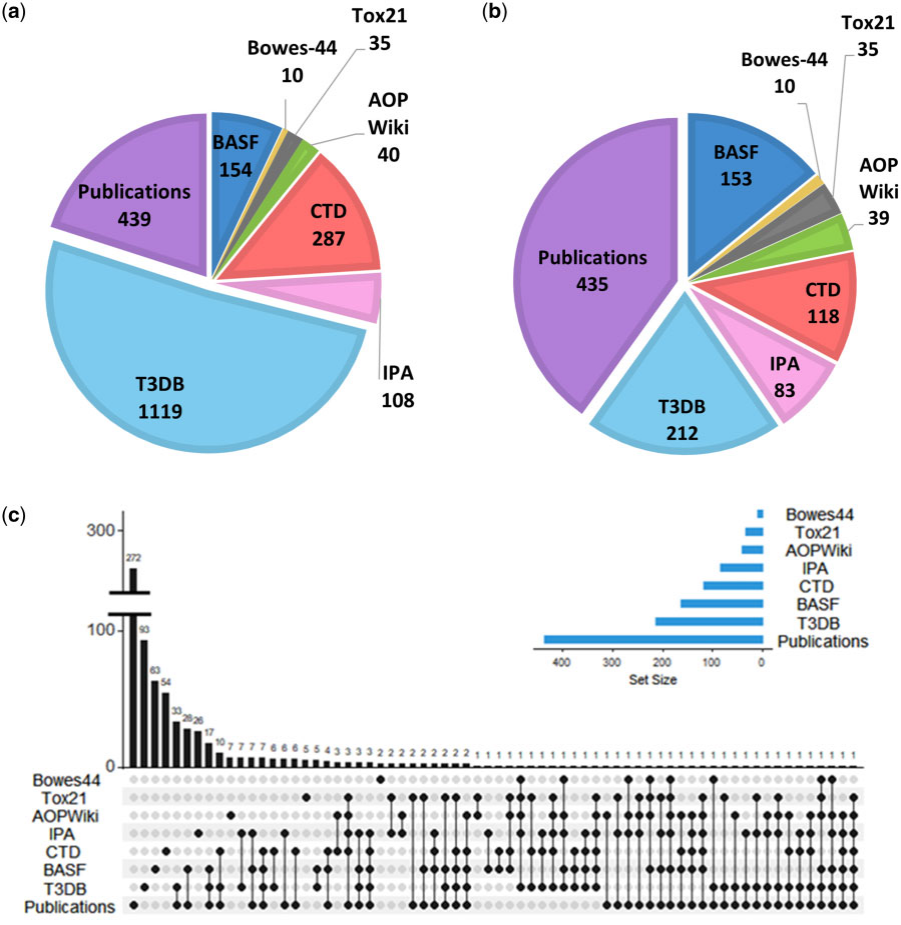
Number of metabolites (A) extracted from each of 8 selected toxicological resources as possible metabolic biomarkers of toxicity (all with Human Metabolome Database IDs); (B) selected for inclusion in the proposed MTox700+ panel following their extraction, filtering, and manual review from 8 resources; (C) intersection of the 722 unique metabolites included in the proposed MTox700+ panel highlighting the importance of extracting knowledge from all 3 types of resources including multiplexed assays (BASF metabolic biomarker panel, Bowes-44 pharmacological biomarker panel, and Tox21 assays), databases (AOP [Adverse Outcome Pathway] Wiki, CTD [Comparative Toxicogenomics Database], IPA [Ingenuity Pathway Analysis knowledgebase], T3DB [Toxin and Toxin Target Database]), and the published literature. Graphical representation was obtained using UpSetR (Lex *et al.*, 2014).

Curation of the BASF assay yielded a total of 202 metabolite entries, of which 21 were labeled as “unknown” in Sperber *et al.* (2019), and a further 27 metabolites lacked a sufficiently specific name to allow their identification, eg, “phosphatidylcholine No. 02” and “TAG (C16:0, C16:1).” Of the remaining 154 metabolite names, 147 were each assigned a unique HMDB ID, whereas 7 of the metabolite names were each assigned multiple possible HMDB IDs. This was necessary as the unsaturated bond configurations for these 7 lipids were unknown; hence, all possible HMDB IDs were retained for ease of filtering and comparison of lists. However, in the figures below, only the unique 154 (147 + 7) metabolites are presented (Figure 4A; see Supplementary Material 1, tab “BASF”). Importing the biomarkers from the Bowes-44 assay resulted in a list of 10 metabolites, all with HMDB IDs (Figure 4A; see Supplementary Material 1, tab “Bowes-44”). Curation of the Tox21 assays yielded a total of 37 metabolite entries, of which 35 were assigned HMDB IDs; 2 entries lacked specificity and could not be identified (Figure 4A; see Supplementary Material 1, tab “Tox21”).

Next, the 4 database resources were interrogated. After performing a manual search of the AOP Wiki, 40 metabolites were extracted and all were assigned HMDB IDs (Figure 4A; see Supplementary Material 1, tab “AOP Wiki”). A total of 601 metabolite entries were extracted from the CTD, of which 287 were assigned HMDB IDs (Figure 4A; see Supplementary Material 1, tab “CTD”). Of the large number of rejected entries majority were xenobiotics, mainly biphenyls, diphenylethers, diphenylmethanes, phthalates, naphthalenes, halogenated, and inorganic compounds. Furthermore, 2 strategies were used to extract relevant metabolite information from the IPA database (described in Extraction, Filtering, and Merging of Metabolite Resource Lists to Create Proposed Metabolic Biomarker Panel—MTox700+ section). The first strategy comprised searching for the SVHCs and submitting the findings to the “Pathway builder” module to assign any molecular associations. Of the 203 SVHC that were searched for by CAS number in the IPA knowledgebase, 71 were present. Of these, IPA’s “Pathway builder” module discovered endogenous metabolic associations for 22 of them, resulting in 68 SVHC-associated metabolites. The second strategy involved inspecting 26 of IPA’s toxicity pathways and extracting the pathway-associated metabolites. Employing the second search approach resulted in 17 of IPA’s toxicity pathways with metabolite associations, corresponding to 77 metabolites. The lists from both search approaches were combined to produce a single IPA resource list of 130 unique metabolites, of which 108 metabolites had HMDB IDs (Figure 4A; see Supplementary Material 1, tab “IPA”). Finally, for the T3DB database, a total of 3533 small molecule entries were identified of which 1119 had HMDB IDs (Figure 4A; see Supplementary Material 1, tab “T3DB”). The majority of the rejected small molecule entries arose from xenobiotics.

The initial query search of published literature using the Abstract Sifter returned 935 papers that were published between 1983 and 2020, and each abstract was examined manually for information on metabolic biomarkers. When the abstract was not sufficiently clear, the publications were studied in greater detail. From the 935 papers examined, 83 (published between 1991 and 2020) were retained. Curation of the retained publications yielded 511 metabolite entries, of which 439 were metabolites with HMDB IDs (Figure 4A; see Supplementary Material 1, tab “Publications”).

Having compiled the 8 metabolite resource lists, all with HMDB IDs, the next step was to filter these against the HMDB/MetaboLights MML. In conjunction with manual inspecting and filtering, this ensured that the proposed biomarkers were human-relevant and that all drugs and xenobiotics were removed. Metabolites from the resource lists that were not present in the MML were individually reviewed to determine their origin, with only human-relevant metabolites retained. Figure 4B shows the number of potential metabolic biomarkers of toxicity, after filtering, for each of the 8 metabolite resource lists.

All 8 resource lists were then combined to produce the proposed MTox700+ panel for toxicology, which comprised a total of 722 unique metabolites (see details in Ranking the Importance of Metabolites in the Proposed MTox700+ Panel section). The metabolite resource lists shared common metabolites, although the extent of overlap was relatively low (Figure 4C) with 525 (73%) metabolites being derived from a single resource. This highlights the importance of combining information from multiple toxicological resources, as reported here for the first time.

#### Associating Disease and/or AOs With Each Proposed Metabolic Biomarker in the MTox700+ Panel

To maximize the confidence in the predictivity of the biomarkers, it was important to attempt to assign 1 or more disease and/or AOs to each of the proposed 722 metabolites. According to information extracted from HMDB, 492 of the proposed metabolic biomarkers are associated with at least 1 disease, based on the disorder classification from OMIM (see Supplementary Material 2 AOs and Pathways, tab “Disease AO ToxFunction”). Interrogating IPA revealed that 178 of the proposed biomarkers are linked to 1 or more “toxicity functions.” A manual search of the AOP Wiki showed that 33 of the proposed metabolic biomarkers (or molecular KEs in this case) are associated with 37 AOs. In addition, curation of publications revealed that 208 of the proposed biomarkers are linked to 1 or more AOs.

**Figure 5.**
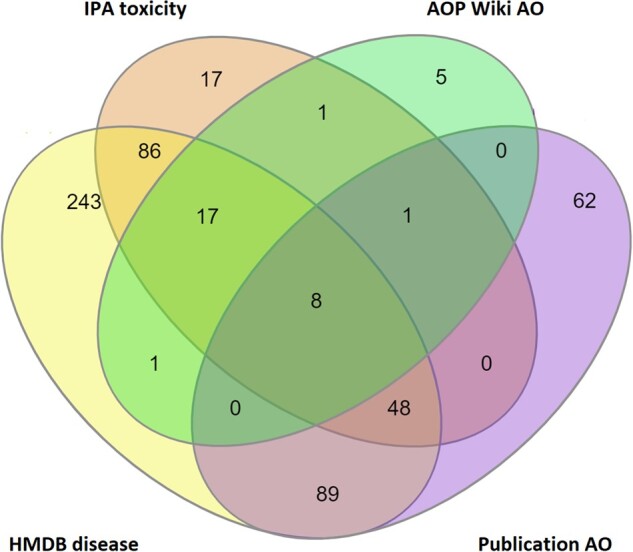
Number of proposed metabolic biomarkers within the proposed MTox700+ panel that are associated with disease and/or adverse outcomes (totaling 578 of 722 possible metabolites), derived from 4 different data sources: HMDB (Human Metabolome Database), IPA (Ingenuity Pathway Analysis), AOP (Adverse Outcome Pathway) Wiki, and published literature.

In total, 80% (578 out of 722) of the proposed metabolic biomarkers are associated with at least 1 disease or AO, with 8 of the proposed biomarkers having a recognized adverse phenotype in all 4 AO sources (Figure 5). Of the 578 metabolites with AO associations, 453 were linked to multiple AOs (with 70% of those metabolites being associated with 10 or fewer AOs), and 125 metabolites were linked to a single AO.

#### Associating Molecular Pathways With Each Proposed Metabolic Biomarker in the MTox700+ Panel

**Figure 6.**
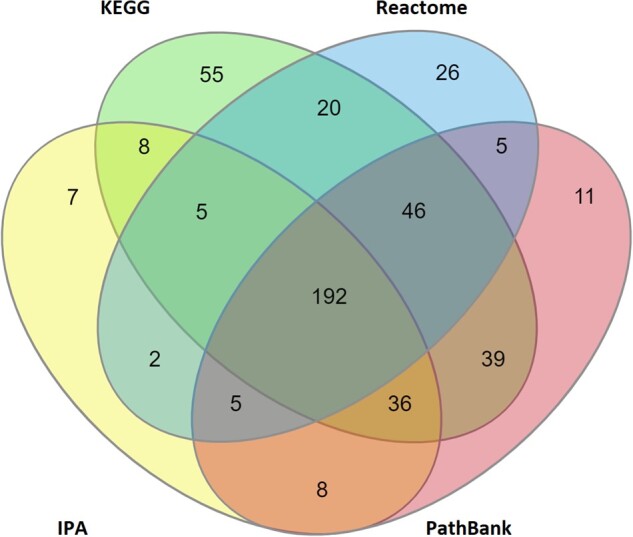
Number of proposed metabolic biomarkers within the proposed MTox700+ panel that are associated with reliable molecular pathways (totaling 465 of 722 possible metabolites), derived from 4 different pathway sources: IPA (Ingenuity Pathway Analysis), KEGG (Kyoto Encyclopedia of Genes and Genomes), Reactome, and PathBank.

To increase the informative value of the biomarkers, it was attempted to assign 1 or more reliable molecular pathways to each of the proposed 722 metabolites (see Supplementary Material 2 AOs and Pathways). Interrogating IPA revealed that 263 of the metabolites are linked to 1 or more canonical pathways. Examining KEGG revealed that 401 of the proposed metabolic biomarkers are associated with at least 1 canonical pathway. The data extracted from Reactome demonstrated that 301 proposed biomarkers are linked to 1 or more pathways. Although at least one of the PathBank pathways was associated with 342 of the metabolites. In total, 64% (465 out of 722) of the proposed metabolic biomarkers are associated with at least 1 molecular pathway, with 192 of these participating in pathways from all 4 pathway sources (Figure 6).

**Figure 7.**
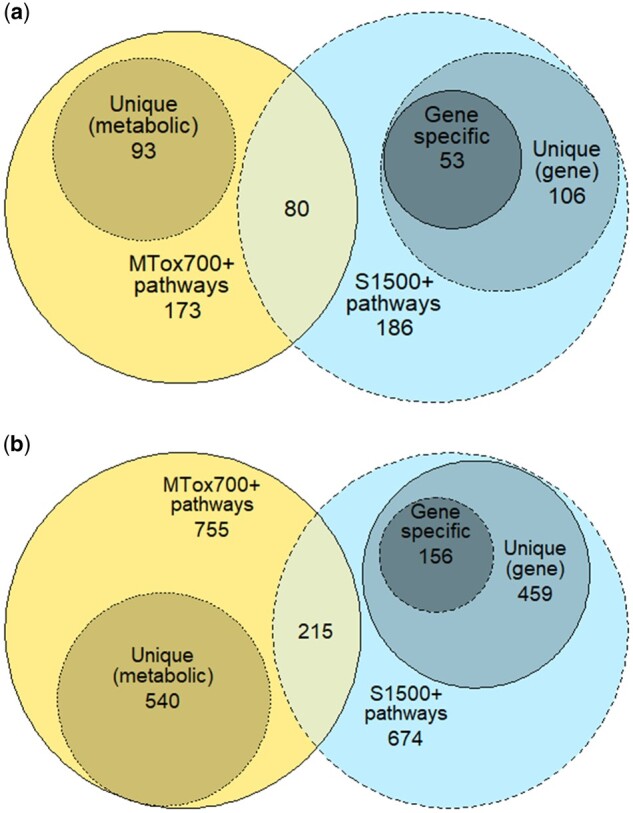
Intersection of molecular pathways associated with MTox700+ panel metabolites and S1500+ panel genes in (A) KEGG (Kyoto Encyclopedia of Genes and Genomes) and (B) Reactome. In both (A) and (B), the center of each diagram represents pathway overlap between the 2 molecular panels (for reliable pathways); the medium-sized circles portray the unique pathways for each panel; and the smallest circle on the right shows pathways with no metabolites.

Next, we sought to address the question whether the measurement of the proposed MTox700+ panel would add value to an experiment that is already applying the S1500+ gene panel. The reliable molecular pathways associated with both panels (MTox700+ and S1500+) were compared, first based on KEGG pathways and then those in Reactome (see Supplementary Material 2, tab “S1500+ pathways”). Metabolite and gene-associated pathways exhibited a moderate overlap in KEGG, with 80 out of 186 S1500+ associated pathways also identified as reliable metabolite-associated pathways (Figure 7A). Of the remaining 106 molecular pathways in S1500+, 53 are gene specific, ie, are not metabolic pathways and therefore do not include any metabolites (a further 37 pathways are associated with metabolic biomarker metabolites; however, these are not “reliable” metabolite-associated pathways, ie, did not meet the minimum threshold of 3 metabolites per pathway). Metabolite and gene-associated pathways overlapped to a lesser extent in Reactome, with 215 out of 674 S1500+ associated pathways also identified as reliable metabolite-associated pathways (Figure 7B). Of the remaining 459 molecular pathways in S1500+, 156 are gene-specific (216 pathways are also associated with metabolic biomarker panel metabolites, but these metabolic biomarkers are not a part of “reliable” metabolite-associated pathways).

Considering the findings from the KEGG and Reactome databases together, the 80 KEGG pathways represented on both molecular panels include 375 MTox700+ panel metabolites, and the 215 common Reactome pathways encompass 277 MTox700+ panel metabolites. In total, 58% (420 unique metabolites out of 722) of the proposed metabolic biomarkers are associated with 295 molecular pathways included in the S1500+ panel. Of particular note is that measurement of the proposed MTox700+ panel would also provide information on many reliable molecular pathways that are not measured by the S1500+ panel, specifically 93 additional pathways in KEGG and 540 pathways in Reactome (see Supplementary Material 2, tab “S1500+ pathways”).

#### Associating Analytical Assays and Reference Standards With Each Proposed Metabolic Biomarker in the MTox700+ Panel

To maximize the likelihood that the proposed MTox700+ panel can be implemented into regulatory testing, the availability of analytical assays and reference standards was assessed (see Supplementary Material 3 Ranked MTox700 panel). Reference standards can serve 2 purposes: first, they are required to achieve the highest level of confidence in metabolite identification, so-called MSI level 1 (Sumner *et al.*, 2007); and they are required if the absolute quantification of metabolites is sought. The levels of analytical confidence in both the identification and quantification of each metabolite measured in metabolomics or targeted metabolite assay will need to be reported according to the OECD Metabolomics Reporting Framework (Harrill *et al.*, 2021). Assay types were sourced from BASF, Bowes-44, Tox21, CTD, and multiple publications. Considering these sources, the majority of metabolites (432) have previously been measured using LC-MS, 93 metabolites detected using GC-MS, a further 164 metabolites were measured using either LC-MS or GC-MS (it was not clarified by BASF), 56 metabolites using NMR spectroscopy, and a further 77 metabolites have been measured using other analytical methods. Many metabolites will be detectable across multiple assays. In summary, 80% (578 out of 722) of the panel metabolites are measurable using at least 1 assay, with LC-MS being the most applicable. Based on our search criteria, reference standards are available for 90% (649 out of 722) of the panel metabolites.

#### Metabolic Biomarker Ranking

Several factors were considered to create a biomarker ranking system that prioritizes metabolites based on the relevance and reliability of each biomarker in the MTox700+ panel. These included the amount of existing information collected from multiple toxicological resources, AO sources, and pathway sources that indicated a metabolite was already used as a biomarker in toxicology; pathway consistency with the S1500+ gene panel; and practical considerations for measuring a metabolite in the laboratory (see Supplementary Material 3).

The first ranking took into account each metabolite’s total coverage of 8 toxicological resources, 4 AO sources, and 4 pathway sources**.** The results indicated that 406 metabolites had limited existing information (scoring below 33%), generally being present in just 1 toxicological resource (typically from recent publications) and containing scarce information from AO sources and/or pathway sources. Higher coverage of resources, AO, and pathway sources was observed for 255 metabolites (medium score of 33–66%), and 61 metabolites scored highly with > 66% coverage in the resources, AO, and pathway sources.

The second ranking considered pathway consistency with S1500+ gene panel, resulting in 420 proposed metabolic biomarkers meeting the criteria.

The third ranking was derived based on the measurement feasibility for each metabolite, including assay and reference standard availability. Both of these were available for the majority of metabolites (515 of 722), with a further 197 metabolites associated with either an assay or a reference standard.

## DISCUSSION

Although the publicly available S1500+ human biomarker panel has helped to drive the application of transcriptomics to predict pathway perturbations (Mav *et al.*, 2018), no equivalent initiative has been reported to develop a metabolic biomarker panel. Yet, the need is great, as it is metabolomics that is capable of measuring downstream molecular phenotypes that more closely relate to adversity. To date, the only metabolic biomarker panel for toxicology is commercial, developed by BASF as a cornerstone of their MetaMapTox database that describes rodent responses to more than a thousand test chemicals (Van Ravenzwaay *et al.*, 2015). The success of BASF’s metabolite panel and database, applied to predict a substance’s MoA and for category formation to support read-across, is evidenced by multiple publications (Sperber *et al.*, 2019; Van Ravenzwaay *et al.*, 2012, 2015). Another toxicology resource—the AOP Wiki, is freely available and hosts some metabolic KE-based AOPs, eg, AOP162 “Enhanced hepatic clearance of thyroid hormones leading to thyroid follicular cell adenomas and carcinomas in the rat and mouse” (Dellarco *et al.*, 2006); however, this resource is still relatively small and few metabolic KEs have been documented. QIAGEN IPA is notable as it contains multiple metabolite associations with pathways and AOs, though it is primarily a biomedical database. Most molecular toxicology resources remain gene/protein focused, with only a few featuring metabolites, highlighting the importance of the information assimilated in this study. Here, a metabolic biomarker panel for toxicology has been proposed by combining knowledge from multiple toxicological resources—including existing multiplexed molecular assays, databases, and the literature.

Several challenges were encountered while developing MTox700+, particularly the lack of consistency in classifying and naming both metabolites and metabolic pathways. First, metabolite repositories rarely delineate between drugs, dietary-derived metabolites, other metabolites arising from environmental exposure (eg, xenobiotics), and endogenous metabolites, all of which can be part of the detectable human metabolome; some resources refer to these simply as “chemicals,” lacking important subclassifications. Even where attempts have been made to define an appropriate ontology (eg, in HMDB; Wishart *et al.*, 2018), there is no single filter that allows, eg, clear distinction between drugs and some endogenous metabolites that are sometimes labeled as drugs (eg, the amino acids L-arginine and L-tryptophan, and some vitamins and hormones). Therefore, metabolites had to be manually curated to resolve these apparent conflicts. The second challenge in assimilating multiple metabolic resources was the lack of standardized names and/or identifiers for metabolites. Although this problem can be alleviated using translation tools (van Iersel *et al.*, 2010; Wohlgemuth *et al.*, 2010), sometimes these tools do not recognize metabolite names/identifiers leading to manual translation of the identifiers. For metabolomics to grow as a tool for assessing chemical hazards, it will be important that study authors define metabolite names and identifiers, as recently proposed in the OECD Metabolomics Reporting Framework (Harrill *et al.*, 2021). A further difficulty encountered was incomplete metabolic names, mainly for lipids, making it impossible to identify some potential biomarkers. Similar to the difficulties encountered for individual metabolites, inconsistent molecular pathway ontologies were also a major issue when working with multiple pathway sources. Despite some pathways bearing the same name in most pathway databases (eg, glycolysis/gluconeogenesis, pyruvate metabolism, sphingolipid metabolism, etc.), the pathway ontologies remain largely inconsistent, ie, similar pathways (in terms of contents and biological function) can have alternative pathway names, additional members/reactions between members, and/or be separated into multiple subpathways. Hence, there is a substantial need to standardize pathway ontology across databases, or minimally to describe the mapping between these resources.

Another challenge arose during the initial searches for the information now contained within the MTox700+ panel due to the relative sparsity of metabolomics data and knowledge in toxicology. For example, in contrast to the well-documented associations of metabolic biomarkers with disease outcomes and/or disease-related molecular pathways (Wishart *et al.*, 2021), metabolite associations with toxicity-specific AOs and pathways are surprisingly rare and were available only from the AOP Wiki, IPA, and some publications. Furthermore, this study revealed that the intersection of metabolites between toxicological resources is low; eg, of the 435 proposed metabolic biomarkers derived from published literature, 273 (63.6%) were not included as putative biomarkers in any of the multiplexed assays or in the database resources. This highlights the considerable importance of recent publications as a source of putative biomarkers, although the depth of investigation of a biomarker in a single publication is typically less rigorous than for biomarkers in the already-established assay panels. This in turn highlights how AO predictions derived from applying the MTox700+ panel could potentially be misinterpreted. Specifically, metabolites that have been extensively researched, such as ATP and cholesterol, are linked to multiple AOs and can serve as more universal biomarkers. However, less studied metabolites that are currently associated with a single AO can be misinterpreted as being highly specific markers linked to an adversity, yet upon further investigation these may also prove to be universal markers. This lack of knowledge could be addressed by the metabolomics community targeting such biomarkers in the MTox700+ panel in future toxicology studies. It is also important to note that a single metabolite is not an adequate representative of an AO or a pathway as it will almost certainly participate in a number of AOs or pathways, hence only a combination of set metabolites is likely be specific for an MoA, and hence better suited to determine adversity.

Similar to the strategy employed here, the 2 main drivers for gene selection in the S1500+ panel were toxicological/pathological relevance and pathway representation (Mav *et al.*, 2018). An important question still to be addressed in the emerging applications of omics technologies to regulatory toxicology is which approach(es) can deliver the minimal mechanistic information required to enable regulatory decision making, eg, whether a combination of upstream transcriptomics and downstream metabolomics is required to define a chemical’s MoA and/or adversity. To support this ongoing discussion, the relationship between the S1500+ and MTox700+ panels was investigated. In addition to multiple overlapping reliable molecular pathways, which could be used in a weight-of-evidence approach to identify the MoA, subsets of both genes and metabolites each participate in unique pathways, suggesting a complementarity of the 2 molecular panels. Similar to the importance of new data for better defining the associations between metabolites and AOs, the generation of new multi-omics datasets that measure both molecular panels will help to inform the community on the relative contributions of transcriptomics and metabolomics to identifying and characterizing hazards.

Although the practical deployment of the MTox700+ panel in toxicology studies is a logical next step, this is not without challenges. As introduced above, a reference standard is required to identify each metabolite with the highest level of confidence (Sumner *et al.*, 2007). Currently, this is not possible as only 646 of the metabolites in the panel are commercially available. Also, not all 722 metabolites will be detectable in all sample types, with subsets of the full panel applicable to different tissues and/or biofluids. For instance, metabolites extracted from the BASF multiplex assay were derived from studies on rat plasma and therefore will primarily be applicable to this sample type. Metabolites derived from the AOP Wiki, CTD, T3DB, and the published literature are applicable to a wider variety of sample types (tissues, biofluids, and/or cells), while the metabolites measured using the Bowes-44 and Tox21 multiplexed assays are of greatest relevance to *in vitro* cell lines.

The toxicological application of the panel may also dictate which subset of metabolites to measure, for instance, hazard identification may prioritize the measurement of metabolites associated with AOs. Irrespective of this granularity, we strongly advocate that the community attempts to measure as many of the MTox700+ metabolites as possible, to identify them confidently, and report their relative quantitative changes in response to chemical exposure, as this will increase the metabolic knowledge associated with the panel and increase its ability to predict downstream biological effects. In particular, quantitative metabolic measurements will be required to distinguish adaptive changes from adverse effects, consistent with the concept of a quantitative AOP (Conolly *et al.*, 2017). By ranking the MTox700+ panel metabolites, it was determined that 316 metabolites (with medium or high total coverage score) have substantial toxicological relevance, and 498 metabolites have an analytical assay and a reference standard available; hence, the barrier to the community adopting at least part of the panel is relatively low and could realize a step-change in the field.

In conclusion, to facilitate the application of metabolomics data in regulatory toxicology, multiple existing toxicological resources have been interrogated—including multiplexed assays, databases, and published literature—to propose a panel of metabolic biomarkers that have the potential to predict MoA and adversity. The creation of the human-relevant MML, comprising 8658 metabolites, was an important step for enabling the management of individual metabolite lists. Selection and subsequent interrogation of the toxicological resources yielded 189 proposed metabolic biomarkers from 3 existing multiplexed assays (BASF, Bowes-44, and Tox21), 346 proposed biomarkers from 4 database resources (AOP Wiki, CTD, IPA, and T3DB), and 435 proposed biomarkers from the literature. Merging all 8 resources generated a list of 722 metabolites, representing a metabolic biomarker panel for toxicology—MTox700+, of which 578 (80%) of the markers are associated with a disease (or toxicity or AO) and 465 (64%) are associated with reliable molecular pathways. Assessing the pathway compatibility between the MTox700+ and S1500+ panels showed that 420 (58%) of the metabolic biomarkers are associated with shared reliable molecular pathways. Through the future use of this panel, it is possible that some metabolites may be removed (if they do not demonstrate sufficient predictivity) while further metabolic biomarkers will be added (discovered via untargeted or hybrid targeted-untargeted metabolomics), hence the MTox700+ panel is predicted to evolve over time. Here, we have launched this metabolic biomarker panel, with the intention to help build foundational knowledge to support the generation of molecular mechanistic data for chemical hazard assessments. 

## SUPPLEMENTARY DATA

Supplementary data are available at *Toxicological Sciences* online.

## DECLARATION OF CONFLICTING INTERESTS

Professors Mark Viant and John Colbourne are employees of the University of Birmingham. They are also Founders and Directors of Michabo Health Science (MHS) Ltd., a spin-out company of the University of Birmingham. MHS also operates as a trading division of University of Birmingham Enterprise Ltd., a wholly owned subsidiary of the University of Birmingham. MHS provides scientific consultancy services in New Approach Methodologies (NAMs) specialising in ‘omics technologies and computational toxicology.

## Supplementary Material

kfac007_Supplementary_Data
